# Extraordinary Case of Hydrocephalus

**Published:** 1852-11

**Authors:** 


*Extraordinary Case of Hydrocephalus.*—A post-mortem examination was made, (by order of the coroner in London, on the 5th of Sept. 1852,) of the body of Joseph Devine, aged fourteen years, found in the Regents Canal, having fallen into the water while being attacked with a fit. The head measured twenty-seven and a quarter inches in circumference; seventeen and a half inches across the top from ear to ear; nineteen and a half inches from the nape of the neck up to the centre of the back, over the crown to the junction of the eyebrows; eleven and three-quarter inches from one parietal bone to the other. The skull was as thin as that of a child two years old, and the sutures were open like those of an infant, never having closed. When punctured, upwards of five imperial pints of water escaped from it, and the substance of the brain itself weighed three and three-quarter pounds. With the exception of the celebrated case of Cardinal, who lived to the age of thirty-two, is the largest head on record.—*Ib.*

